# Drug-based perturbation screen uncovers synergistic drug combinations in Burkitt lymphoma

**DOI:** 10.1038/s41598-018-30509-3

**Published:** 2018-08-13

**Authors:** K. Tomska, R. Kurilov, K. S. Lee, J. Hüllein, M. Lukas, L. Sellner, T. Walther, L. Wagner, M. Oleś, B. Brors, W. Huber, T. Zenz

**Affiliations:** 1 Molecular Therapy in Haematology and Oncology & Department of Translational Oncology, NCT and DKFZ, Heidelberg, Germany; 2 Division of Applied Bioinformatics, DKFZ, Heidelberg, Germany; 3 Faculty of Biosciences, Heidelberg University, Heidelberg, Germany; 4 Genome Biology Unit, EMBL, Heidelberg, Germany; 5 Department of Medicine V, University Hospital Heidelberg, Heidelberg, Germany; 6 Dept. of Hematology, University Hospital and University of Zurich, Zurich, Switzerland

## Abstract

Burkitt lymphoma (BL) is a highly aggressive B-cell lymphoma associated with MYC translocation. Here, we describe drug response profiling of 42 blood cancer cell lines including 17 BL to 32 drugs targeting key cancer pathways and provide a systematic study of drug combinations in BL cell lines. Based on drug response, we identified cell line specific sensitivities, i.e. to venetoclax driven by BCL2 overexpression and partitioned subsets of BL driven by response to kinase inhibitors. In the combination screen, including BET, BTK and PI3K inhibitors, we identified synergistic combinations of PI3K and BTK inhibition with drugs targeting Akt, mTOR, BET and doxorubicin. A detailed comparison of PI3K and BTKi combinations identified subtle differences, in line with convergent pathway activity. Most synergistic combinations were identified for the BET inhibitor OTX015, which showed synergistic effects for 41% of combinations including inhibitors of PI3K/AKT/mTOR signalling. The strongest synergy was observed for the combination of the CDK 2/7/9 inhibitor SNS032 and OTX015. Our data provide a landscape of drug combination effects in BL and suggest that targeting CDK and BET could provide a novel vulnerability of BL.

## Introduction

Burkitt’s lymphoma (BL) is a highly aggressive non-Hodgkin lymphoma (NHL), which is driven by the characteristic translocation of the MYC oncogene^1,2^. Gene mutations in BL target essential cancer pathways including e.g. p53^3^, the SWI/SNF complex^4^ and the transcription factor TCF3 (E2A) or its negative regulator ID3. Pro-survival signals are elicited through phosphatidylinositol-3-OH kinase pathway (PI3K) activation by TCF3/ID3 mutations and tonic B-cell receptor signalling^5,6^.

BL can be managed very effectively using intensive chemoimmunotherapy, especially in younger patients^7,8^. Current treatment of BL consists in intensive chemotherapy including combinations of cyclophosphamide, doxorubicin, methotrexate, vincristine and prednisone or combinations of methotrexate, cytarabine, etoposide, ifosfamide and dexamethasone^7^. Chemotherapy of BL has been successfully combined with the CD20 antibody rituximab.

However, the elderly and patients with immunodeficiency have an inferior outcome^7^, which underscores the necessity for alternative treatments. These are unlikely to emerge from further chemotherapy optimization. Relapsed or refractory BL has a dismal prognosis and is generally considered incurable. Therefore, platforms to generate rational novel combinations for BL could have immediate clinical consequences and may allow a functional dissection of genotype specific sensitivities. Cell lines provide a robust model for drug response studies and can be used to develop new treatment strategies including combinations. Recent comprehensive large-scale studies provided detailed analysis of tumour specific determinants of drug response based on molecular characterization of cell lines and their pharmacological profiles^9–12^. Pharmacological profiling studies identified synergistic drug combinations with ibrutinib in activated B-cell-like diffuse large B-cell lymphoma (ABC DLBCL)^13,14^ or NF-κB-targeted strategies in mantle cell lymphoma (MCL)^15^. While previous studies include a large number of cell lines, individual entities were underrepresented, i.e. the number of BL cell lines ranges from as few as 3 up to 11 in the mentioned platforms.

Previous *in vitro* studies revealed synergistic drug interactions i.e. between PI3K inhibitor and chemotherapy^16^ as well as mTOR and histone deacetylase inhibitors^17^. However, currently there are no synergistic combinations of targeted drugs in clinical use, hence arises the necessity for preclinical models to provide rational drug combinations.

Recent studies provide evidence for dependency of BL on tonic B-cell receptor (BCR) signalling to PI3K^18^. While activation of MYC in mouse B cells was insufficient for lymphomagenesis, a cooperating mechanism of PI3K activation in BL was identified^19^.

BET family, including BRD2, BRD3, BRD4 and BRDT, influences gene expression by recruiting transcriptional regulators to specific genomic locations^20,21^. BRD4 plays an important role in transcription of many genes including *MYC*^22^, which is overexpressed in BL, and hence BET inhibition potently suppresses the expression of *MYC* in leukaemia and lymphoma cell lines leading to induction of cell cycle arrest and apoptosis^21^.

Here, we describe a detailed study of drug response and combination treatments across a panel of haematological malignancy derived cell lines focusing on BL. We identify a subgroup of BL lines responsive to PI3K and BCR pathway inhibition and delineate numerous cooperative interactions of PI3K/AKT/mTOR pathway and BET inhibition. Strong synergy between BET and cyclin dependent kinase (CDK) inhibition by SNS-032 provides a rational for clinical testing of this combination.

## Results

### Drug response phenotypes of blood cancer models

**Figure 1:**
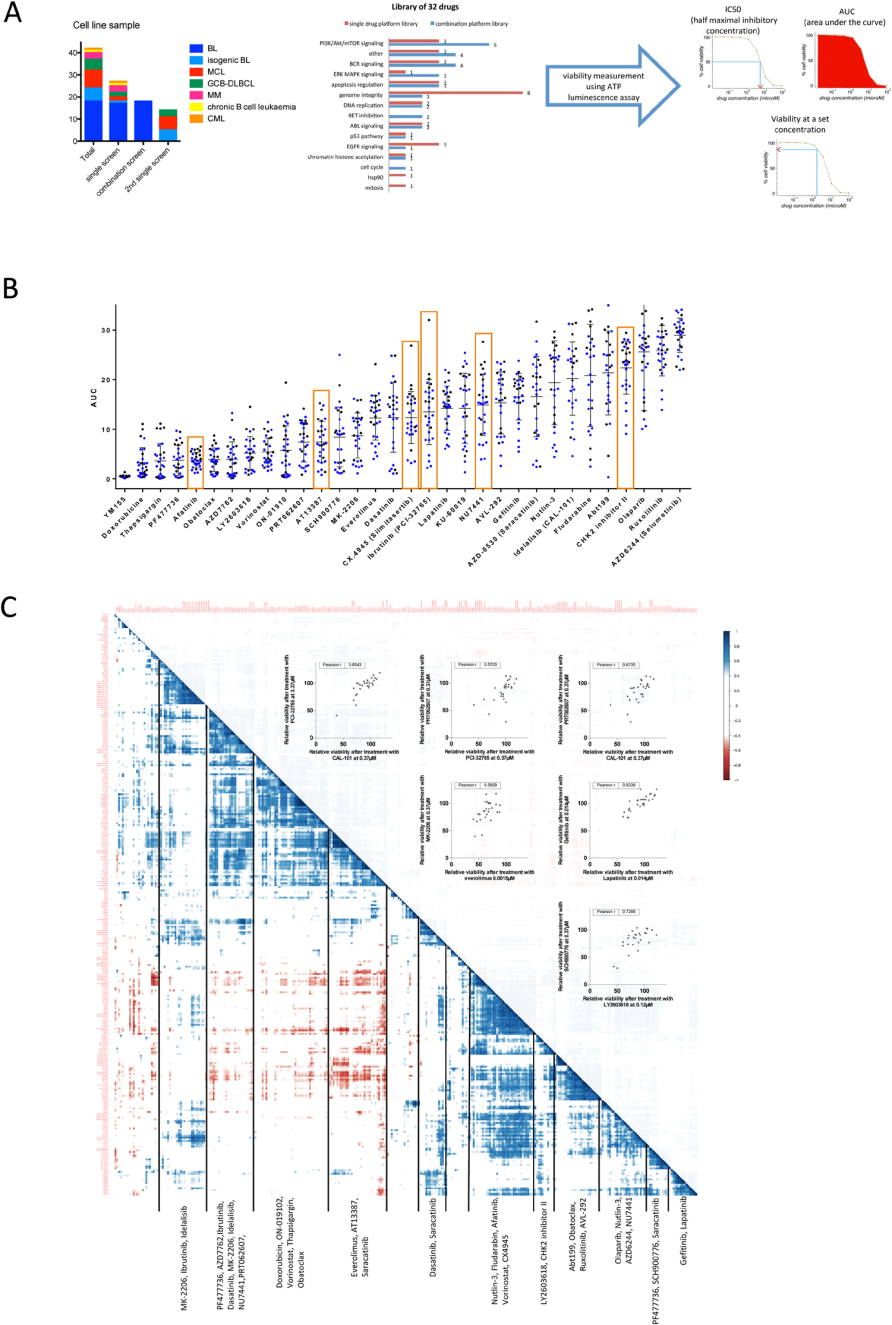
Mapping drug response in blood cancer cell lines. (**A**) Screen of drug effects on a panel of blood cancer cell line models from different disease entities. The screens included 32 drugs at 10 concentrations, which target different pathways^12^. For the combination screen on BL (n = 17) ibrutinib, idelalisib and OTX015 were added. Viability was assessed at 48 h by ATP measurements and to avoid confounding based on differences in viability effects of the drugs, we used multiple dose-response surrogates (IC50, AUC) and individual viabilities. (**B**) Drugs lead to variable viability effects across cell lines. Summary of AUC values for 32 drugs across n = 27 cell lines (blue: BL, black other; median and interquartile range shown by bars). Compounds with AUC < 10 were considered toxic. Orange boxes mark drugs that were significantly more effective in BL compared to other cell lines, as indicated by a p-value < 0.01 calculated using an unpaired t-test. Heterogeneity of response across cell lines as well as individual outliers, i.e. in response to dasatinib was observed. (**C**) Heatmap of drug-drug correlations based on single agent viability effects in 27 cell lines is shown (blue represents positive correlation, red represents negative correlation.). Clustering is driven by multiple concentrations of individual drugs (e.g. everolimus, YM155, thapsigargin, AZD6244) as well as inhibitors acting within the same pathway (BCR, PI3K pathways, Chk inhibitors, EGFR inhibitors, Bcl-2 family inhibitors). Scatter plots inlay provides correlation of individual drugs mentioned above.

To identify molecular dependencies and potential therapeutic targets in BL, we measured the effect of 32 drugs in 10 concentrations on the viability of 42 blood cancer cell lines, including 17 BL cell lines, and 6 isogenic BL lines with targeted deletion of p53, SYK, BTK, BLK or CD20 (Fig. 1A). In line with prior cell line screening efforts, we used ATP assessment as a surrogate of viability^23,24^.

Because of the potential bias introduced by response surrogates, we considered IC50, AUC and individual doses (Figs 1A, S1). We observed variable effects on viability with compounds, which were chosen based on their clinical use (i.e. doxorubicin, fludarabine) and targeting of essential cancer pathways (i.e. p53, mTOR, PI3K, AKT, BTK, BCR/ABL) (Figs 1B, S1). We identified drugs that were significantly more toxic in BL compared to other entities (p < 0.01) including inhibitors of BTK (ibrutinib), CK2 (CX-4945), DNA-PK (NU7441), EGFR (afatinib) and CHK2 (Fig. 1B).

To understand the relation of effects produced by different agents, we analysed drug-drug correlations across concentrations. This matrix uncovered a number of highly correlated drugs driven by both multiple concentrations of individual compounds as well as drugs targeting overlapping pathways or nodes of the same pathway. For example, strong positive correlations were shown for multiple concentrations of everolimus (median correlation coefficient *r* = 0.93), venetoclax (median *r* = 0.81) or thapsigargin (median *r* = 0.85; Fig. 1C), a finding that supports the platform consistency. Strong positive correlations were observed for gefitinib and lapatinib, known EGFR pathway inhibitors (*r* = 0.83, Fig. 1C), and the BCR-ABL/SRC inhibitors saracatinib and dasatinib (*r* = 0.89).

Idelalisib (PI3K), everolimus (mTOR) and MK-2206 (AKT) effects were highly correlated as the drugs target members of the PI3K pathway. In line with the known role of SYK, PI3K and BTK in BCR signalling, ibrutinib (BTK) and PRT062607 (SYK) (Fig. 1C) and idelalisib (PI3K) and PRT062607 (SYK) were correlated. In addition, we found multiple drugs to be included in this cluster driven by BCR inhibition including AT13387, an inhibitor of hsp90, which has been shown to be a chaperone required for SYK stabilization in BL^25^. Dasatinib and saracatinib (Src family), the CHK inhibitor AZD7762, and the DNA-PK inhibitor NU7441, which has also been shown to inhibit PI3K and mTOR^26^ also showed similar response across cell lines.

In summary, the analysis of drug-drug associations allowed us to provide evidence for the coherence of the screening platform as well as to provide insight into the pathways involved in viability effects of drugs.

### Drug response heterogeneity in blood cancer

**Figure 2:**
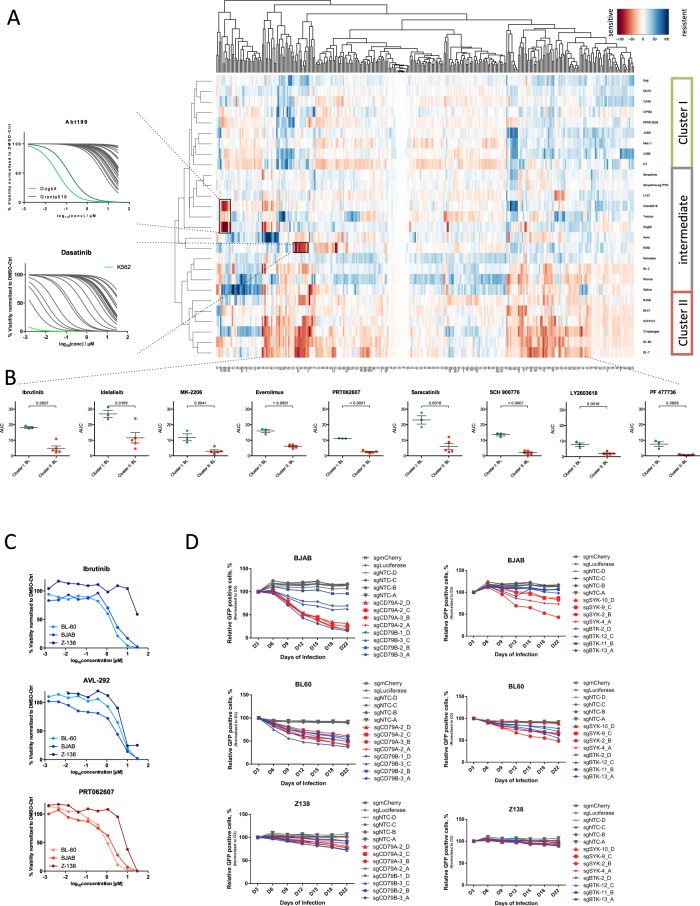
Drug response in blood cancer. (**A**) Unsupervised clustering of cell lines and drugs across concentrations based on viabilities identifies drug response groups. Cluster I consists of cell lines resistant to kinase inhibitors. Cluster II includes cell lines specifically sensitive to PI3K/AKT/mTOR pathway (idelalisib, MK-2206, everolimus), BCR pathway (ibrutinib, spebrutinib, PRT062607), BCR-ABL (dasatinib, saracatinib), hsp90 (AT13387), and Chk inhibitors. The intermediate group is characterized by individual response phenotypes, i.e. a venetoclax sensitive cell lines (Granta-519, DOGKIT) and the CML cell line K562 sensitive to dasatinib and saracatinib, targeting BCR-ABL (see IC50 inlay). (**B**) Comparison of AUC values for selected drugs shows significant differences in sensitivity to ibrutinib, idelalisib, everolimus, MK-2206, PRT062607, saracatinib, SCH900776, LY2603618 and PF 477736 between Cluster I and II. P-values were generated using an unpaired t-test. (**C**) Cell line dependent response to BCR inhibitors shown by exemplary dose-response curves for BJAB, BL-60 (sensitive), and Z138 (resistant). (**D**) To confirm dependency of the sensitive cell lines on BCR signalling we used orthogonal technology (CRISPR-Cas9). A competition assay after silencing of CD79A, CD79B, BTK and SYK shows stronger viability effects on the SYK-inhibitor-sensitive cell lines BL-60 and BJAB, while the viability of the more resistant line Z138 is affected less over time.

We clustered cell lines based on the effect on viability for all drugs and concentrations (Fig. 2A, Supp. Fig. S2) and identified distinct response profiles. Cluster I contained cell lines resistant to multiple drugs including inhibitors of the PI3K and BCR pathways (ibrutinib, spebrutinib, idelalisib, MK-2206, PRT062607 and everolimus) (Fig. 2B, Supp. Fig. S2). The cluster consisted of resistant BL and myeloma (MM) cell lines, in line with BCR independent survival signals in myeloma.

The second response group (Cluster II) showed the strongest response to kinase inhibitors including PI3K inhibitor, as well as a number of other targeted drugs including SRC inhibitors (dasatinib, saracatinib). The dependence of those cell lines on BCR and SYK was validated by knocking out BTK, SYK and CD79A/B using CRISPR/Cas9. The effect of gene knock-out followed the pattern of drug sensitivity and showed that the cell line with highest IC50 values for BTK and SYK inhibition (Z138) was also the least affected by the knockout of CD79A, CD79B and SYK compared to BJAB and BL-60 (Fig. 2C,D). These results provided evidence that drug toxicity is due to on-target effects of the SYK inhibitor and confirmed the above observations with orthogonal technology. The response to BTK inhibitors correlated with a moderate effect of the BTK knock-out on BJAB and BL-60, in line with the survival signals in BL and GCB-DLBCL stemming from BCR signalling.

The intermediate group showed heterogeneous response and included cell lines with individual sensitivities. These were in part driven by specific genotype context, i.e. the CML cell line K562 was sensitive to BCR-ABL inhibitor dasatinib and the Src-family inhibitor saracatinib (Fig. 2A). Increased sensitivity to the bcl-2 inhibitor venetoclax was found in a subgroup of MCL lines with high BCL2 expression (Maver-1, Mino, HBL-2)^27–29^, a subgroup of BCL-2 dependent lymphoma lines (OCI-LY-1 and OCI-LY-8)^30–32^, a BL cell line (DOGKIT) known to harbour BCL2 rearrangements^33^ and the B-NHL line Granta-519, which has been shown to overexpress BCL2^34^ (Figs 2A, S2). The isogenic cell line models generated by CRISPR/Cas9 targeting *TP53 (*sgTP53-Séraphine) clustered with its parental line (*r* = 0.97, Figs 2, S3). It also showed increased resistance to Nutlin-3 and doxorubicin but not fludarabine (Fig. S3).

Combined, these data suggest that the platform was able to uncover both individual sensitivity patterns as well as provide a functional grouping of BL based on response to pathway inhibitors.

### Identification of rational drug combinations in BL

Based on the comprehensive single agent data, we designed a platform to evaluate drug combination effects in BL. We used an optimized library of 32 agents (Fig. 1A) enriched for inhibitors of the PI3K/AKT/mTOR pathway and BET, based on the known role of tonic BCR signalling^18^ and MYC in the disease. To categorize combinations as synergistic, additive or antagonistic and quantify combination behaviour, we applied Chou’s combination index (CI) analysis^35^. We classified combinations as synergistic for CI values below 0.85. Values between 0.85 and 1.15 indicate additive effects, and CI above 1.15 points was assigned antagonistic. The use of the CI analysis has its limitations, e.g. in the context of very toxic combinations or if the drug itself had a very small IC50 value, based on the logarithmic transformation used in the model^36^. Therefore, we also assessed drug interactions using dose-response curves of relative viabilities (Figs S6, S7).

**Figure 3:**
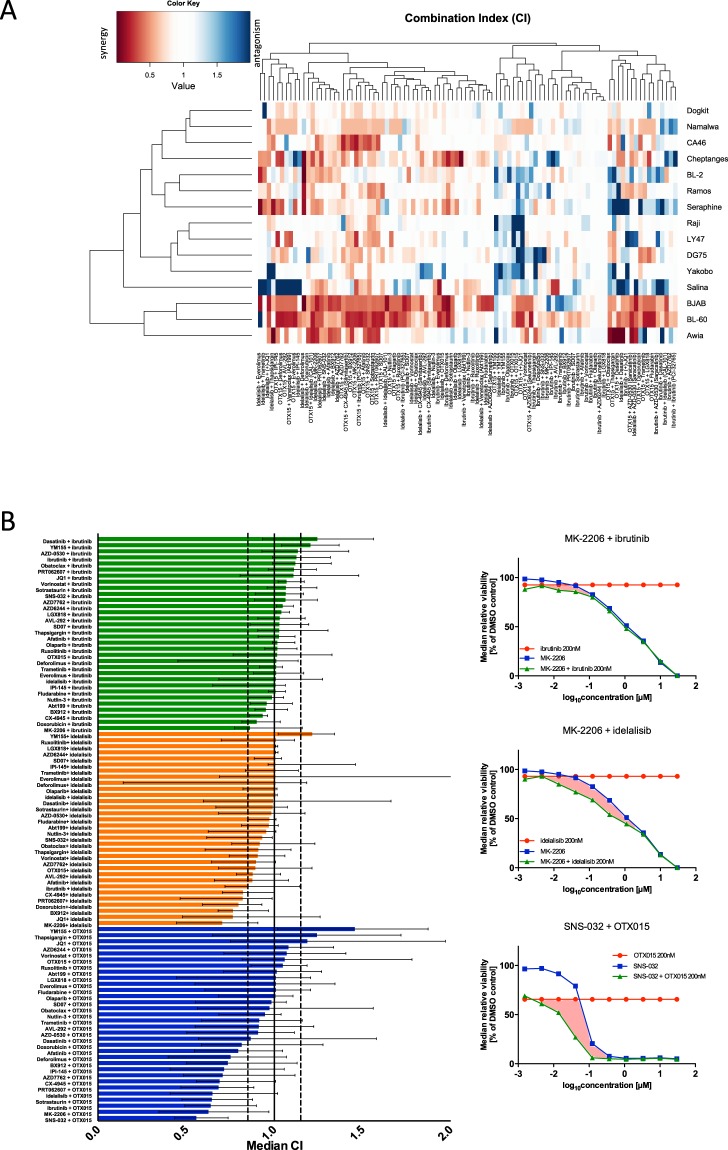
Landscape of synergistic drug combinations in BL. (**A**) Heatmap shows clustering based on CI across cell lines (n = 15). We observed cell lines with dominant combination effects (BJAB and BL-60), as well as drug combinations leading to synergistic effects across cell lines, i.e. deforolimus and idelalisib or SNS-032 and OTX015. Multiple combinations showed cell line dependent effects where combinations lead to both synergistic and antagonistic effects (e.g. Salina, Séraphine). CI values for Gumbus, BL-7 and BL-41 were excluded from the analysis due to high toxicity of the combination drugs, which resulted in unreliable determination of drug interactions. (**B**) Bar plot summarizes median CIs across 18 cell lines for all 96 combinations tested in the combination assay reveal synergistic combinations. Dashed lines correspond to the definitions of drug interaction (<0.85 = synergistic; >1.15 = antagonistic). Error bars indicate interquartile range. The analysis yields few antagonistic and 20 synergistic combinations. Most synergistic combinations were identified for OTX015. (**C**) Median dose-response curves for combinations with the lowest CI: MK-2206 with ibrutinib; MK-2206 with idelalisib and SNS-032 with OTX015 (n = 18 cell lines). The marked area between the combination curve (green) and single agent curves (blue, red) indicates the drug interaction.

We clustered the CI values to assemble an overview of drug combination effects in BL. We found combinations with cooperative effects across almost all cell lines (Fig. 3A). Those included most combinations with OTX015 (with BCR, PI3K, Akt, CDK2/7/9 and doxorubicin) and individual combinations with idelalisib. While most combinations showed additive or synergistic effects, we also identified antagonistic combinations, including combinations of BETi (JQ1 and OTX015) and BCR inhibition combinations with the Survivin inhibitor (YM155). BJAB and BL-60, both BL lines from cluster II (Fig. 2A), showed the highest number of synergistic drug interactions.

We used the median CI as a measure of drug interaction across all BL cell lines. Combinations with OTX015 yielded most synergistic combinations (13/32). Six of 32 idelalisib combinations were synergistic, while for none of the ibrutinib combination CI were below 0.85 (Fig. 3B). The AKT inhibitor MK-2206, resulted in cooperative interactions with ibrutinib, idelalisib and OTX015 with a median CI across BL lines of 0.86, 0.70 and 0.63 respectively (Fig. 3B).

### Ibrutinib combination effects in BL

Most drug combinations with ibrutinib were additive (Fig. 3B), which may be explained by the moderate efficacy of this inhibitor in BL. The lowest CIs were observed for the combinations of ibrutinib with the AKT inhibitor MK 2206, and the chemotherapeutic doxorubicin. We observed similar CI values for combination of ibrutinib with both PI3K inhibitors (idelalisib and IPI-145) and both mTOR inhibitors (everolimus, deforolimus) included in the screen. Hence, additivity between the inhibitor of BCR proximal kinase and inhibitors of PI3K pathway in an entity with tonic BCR signalling was identified. Doxorubicin has also been shown to be an effective combinations partner for ibrutinib as previously shown for other haematological malignancies^11^. Bcl-2 inhibtiors, obatoclax and venetoclax, combined with ibrutinib showed median CI values off 1.12 and 0.96 respectively, which was explained by differential sensitivity to the drugs (Fig. 1B).

### Idelalisib combination effects in BL

Idelalisib was synergistic when combined with inhibitors of BET (JQ1), chemotherapeutics, the AKT inhibitor (MK-2206), a downstream node of PI3K pathway, and the SYK inhibitor PRT062607 with corresponding median CIs of 0.76 (JQ1), 0.80 (doxorubicin), 0.70 (MK-2206), 0.82 (PRT062607). This suggests a positive influence of combining more than one inhibitor of the PI3K pathway, as well as combining inhibitors of BCR proximal kinase SYK and PI3K. These data also provide a rationale for combinations of idelalisib with doxorubicin, which is used in BL treatment regimens.

Although we observed few differences between the response patterns to ibrutinib and idelalisib (Fig. S4), we found idelalisib to be more active in BL than ibrutinib a finding in line with the role of tonic BCR signalling in BL^18^.

### BET inhibitor combination effects in BL

We observed multiple strong synergistic effects for the BET inhibitor OTX015. The lowest CI scores were found for the CDK2/7/9 inhibitor SNS-032, inhibitors of the PI3K/AKT/mTOR and BCR pathway inhibitors. Median CI scores across 18 cell lines ranged from 0.56 (SNS-032) to 0.81 (doxorubicin). Combinations of OTX015 with thapsigargin and YM155 yielded CIs of 1.24 and 1.46, suggesting that these combinations were antagonistic. We analysed the individual dose-response curves and found a subset of cell lines, which showed cooperative effects for this combination (Namalwa, DG75, Gumbus), while in 7 out of 18 lines the interaction between OTX015 and YM155 was antagonistic. Cell lines with lower OTX015 sensitivity showed higher CI values (Fig. S7), suggesting that drug interaction depends on the response to OTX015 rather than YM155. Also, among the combinations of the drug library with OTX015 a much higher variability of drug interaction potentials was found compared to idelalisib an ibrutinib. This is in line with the strong efficacy of this BET inhibitor in BL and its multimodal effect on gene expression.

### BET inhibition shows synergy with PI3K/AKT/mTOR signalling pathway inhibition

**Figure 4:**
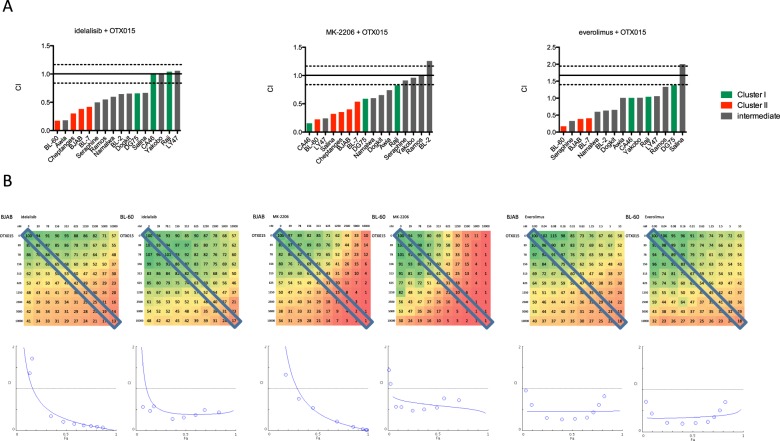
BET and PI3K inhibition are synergistic in BL. (**A**) Boxplots of CI show drug interactions of PI3K inhibitors (idelalisib), the AKT inhibitor MK-2206 and mTOR inhibitor (everolimus). CI could not be calculated for all cell lines (cell lines with values <2 are shown). (**B**) Relative viability (%) data in a 10 × 10 matrix experiment (mean of duplicate values) for combinations of OTX015 with idelalisib, MK-2206 and everolimus. Drugs and respective concentrations are listed on x and y axes. The effect on viability is colour coded (green viability 100%, red 1%). The diagonal box marks viability data used for constant-concentration-ratio analysis of drug interaction, which resulted in the Fa-CI plots (Fraction affected combination index^35^). Fa-CI plot shows CI on y-axis as a function of effect level of the drug combination (Fa; fraction of dead cells compared to control) on x-axis. Data points below the horizontal line (CI = 1) indicate synergy.

We identified a striking number of synergistic interactions between inhibitors of the PI3K/AKT/mTOR signalling pathway, which has been shown to be essential to survival of BL cells^5^, and OTX015 within a subgroup of BL cell lines. Specifically, the PI3K inhibitors idelalisib and IPI-145 (duvelisib), the allosteric AKT inhibitor MK-2206 and the mTOR inhibitors everolimus and deforolimus (Fig. 4A) were found to have synergistic effects with OTX015 (CI 0.15–0.85). As these findings could potentially be limited by the use of a single combination drug concentration, we tested the interactions between BET inhibition and PI3K pathway inhibition by idelalisib, MK-2206 and everolimus in a 10 × 10 dose matrix. The analysis of CI as a function of fraction of cells affected by the drug (CI-Fa plot analysis) showed synergistic interaction for drug combinations of idelalisib (PI3K) and OTX015 with CI_50_ of 0.27 and 0.59 for BJAB and BL-60 respectively (Fig. 4B). The combination of MK-2206 (AKT) and OTX015 was also synergistic in BJAB and BL-60 with corresponding CI_50_ of 0.42 and 0.73. 10 × 10 analysis of the combination of everolimus (mTOR) and OTX015 showed a CI_50_ of 0.46 and 0.35 in BJAB and BL-60 respectively, pointing towards strong synergy. The concave gradient of viability heatmap of the 10 × 10 combination matrix also indicated these cooperative interactions (Fig. 4B).

Importantly, synergy for OTX015 with idelalisib, MK-2206 and everolimus was observed at concentrations *in vitro* that can be safely administered in the clinical setting *in vivo*^37–42^.

### The combination of CDK inhibitor SNS-032 and BET inhibitor OTX015 is synergistic

**Figure 5:**
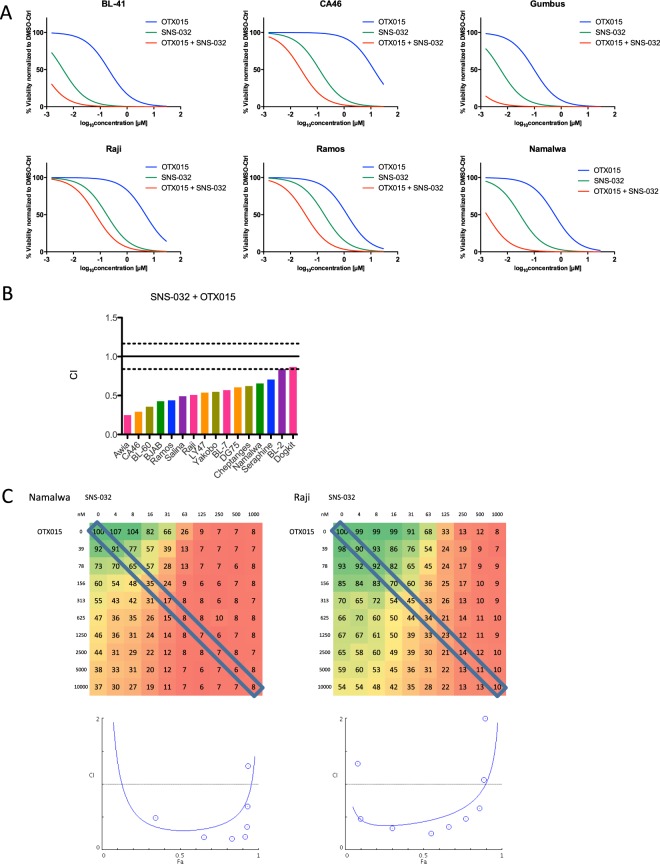
BET and CDK2,7 and 9 inhibition work synergistically in BL. (**A**) Relative viability data indicates synergistic drug interactions between SNS-032 and OTX015. IC50 curves for OTX015 (blue) and SNS-032 (green) as single agents show lower toxicity compared to the IC50 of the combination of SNS-032 (in 10 concentrations) and OTX015 at 200 nM (red). (**B**) Boxplot of combination indices for SNS-032 and OTX015 in 18 cell lines. (**C**) Relative viability (%) data in a 10 × 10 matrix experiment (mean of duplicate values) for combinations of OTX015 with SNS-032. Drugs and respective concentrations are listed on x and y-axis. The effect on viability is colour coded (green high viability, red low viability). The diagonal box marks viability data used for constant-concentration-ratio analysis of drug interaction, which resulted in the Fa-CI plots (Fraction affected combination index^35^). We confirmed synergy in Namalwa and Raji using viability gradient in a 10 × 10 matrix and Fa-CI plots. For Namalwa most combination index values lay below the CI = 1 line, which indicates dose-dependent synergy of the combination of OTX015 and SNS-032. For Raji all data points lie below the horizontal line, which indicates strong synergy.

Our screen revealed the lowest CI for the SNS-032 and OTX015 combinations targeting CDK2/7/9 and BET. Synergistic interaction of the combination of BET inhibition with CDK inhibition was observed in 16 out of 18 cell lines (Fig. 5B). The CDK inhibitor SNS-032 and the BET inhibitor OTX015 were both effective in killing BL lines (Fig. 5A). Synergy was confirmed by the 10 × 10 validation study. The CI-Fa plot analysis (Fig. 5C) showed strong synergistic interaction for drug combination of SNS-32 and OTX015 with CI_50_ of 0.29 and 0.43 in Namalwa and Raji respectively. Also for SNS-032 and OTX015, we observe synergy at concentrations that are achievable in the clinical setting^41–43^.

## Discussion

Therapeutic strategies in oncology undergo dynamic changes, so that there is a need for efficient methods to identify and evaluate new therapeutic targets and define effective drug combinations. Recent large-scale pharmacogenomics studies have proven useful in achieving that goal^9–13,16,17^. In the current report, we demonstrate pharmacological vulnerabilities in BL and uncover effective drug combinations.

The current report provides a comprehensive drug-sensitivity profile of BL cell lines, which have been underrepresented in the previous drug response profiling studies^9,10,13,16,17^.

The platform uncovered known vulnerabilities (i.e. dasatinib in CML, HSP90 inhnhibitor in BL^25^) in parallel to uncovering new targets within a Burkitt lymphoma set (BTK and SYK in a subset of BL).

We report a number of novel cooperative drug interactions. OTX015 (BETi) showed strong combination activity with the highest number of compounds targeting different pathways in nearly all tested models. Synergistic combinations with idelalisib and ibrutinib were found in a subset of cell lines for a smaller set of drugs. Current clinical trials of OTX015 in non-leukaemia haematological malignancies have shown relatively good tolerance of the drug, with low-grade adverse events of anaemia, neutropenia, diarrhoea, fatigue and nausea; high-grade thrombocytopenia was also observed^41,44^. Hence, it could be considered for clinical trials in several combination described in this work, including ibrutinib and idelalisib.

A high number of cooperative drug interactions with OTX015 were found with multiple drugs inhibiting the PI3K pathway, including the PI3K inhibitors idelalisib and IPI-154, the AKT inhibitor MK-2206 and mTOR inhibitors everolimus and deforolimus. Those synergies are in line with the oncogenic signalling activated by tonic BCR signalling^5,19^. Also, it has been suggested that BET inhibition sensitizes PI3K and MYC driven cancer cells to PI3K inhibitor by blocking PI3K pathway reactivation caused by induction and activation of receptor tyrosine kinases (RTKs) through blocking the expression of RTKs^45^. Synergistic interactions between the PI3K pathway inhibitor and BETi have been previously described *in vitro* in other entities including haematological malignancies MCL^46^ and ABC-DLBCL^14^ as well as in solid malignancies including glioblastoma^47^, osteosarcoma^48^ and breast cancer^45^, which underscores the necessity of evaluation of this combination in clinical trials. Interestingly, dual-activity PI3K-BRD4 inhibitors have been recently developed and are tested in clinical trials for hepatocellular carcinoma^49^.

The current study uncovers a novel synergistic combination in BL, a specific CDK2,7,9 inhibitor SNS-032 with BET inhibitor OTX015. This exact mechanism of this combination activity is currently unclear. The targets of both drugs, cyclin-dependent kinase 9 (CDK9) and Brd4, act as positive regulators of p-TEFb^50^, which consists, among others, of CDK9 and cyclin T, and plays a crucial role in activation of transcription. Another possibility is that a Brd4-independent mechanism may recruit or retain P-TEFb and lead to elevated MYC transcription^22^, which in turn can be down-regulated by CDK9 inhibition by dinaciblib in BL cell lines^51^. However, it has to be noted that dinaciclib is not as target-specific as SNS-032. SNS-032 targets not only CDK9, leading to inhibition of transcription, but also CDK7 and CDK2, whose inhibition can block cell cycle progression^52,53^.

This study shows that *in vitro* pharmacological profiling in cell lines is an excellent tool of preclinical evaluation of drug combinations. The dataset provided can function as a training set to predict synergistic drug interactions on a larger scale^54^ and help plan future evaluation of drug combinations. The possibility of *in silico* modelling of predicting drug resistance, drug-target interaction and potential synergistic drug combinations on a large scale based on current drug target and pharmacological profiling databases^54–57^ has been extensively discussed as a relevant alternative drug discovery. Information from such models could help develope efficient *in vitro* experimental setups in the context of developing rational combination therapy.

## Methods

### Cell culture

Cell lines were obtained from G. M. Lenoir (IARC, Lyon, France), A. Rickinson (University of Birmingham, UK) or the DSMZ (Braunschweig, Germany). p53-deficient BL line Séraphine was generated and provided by J. Hüllein (NCT, Heidelberg, Germany). MCL lines (Z-138, Maver-1, UPN-1, HBL-2, Mino, Rec-1) were kindly provided by University of Frankfurt and University of Würzburg. KS. Lee (NCT, Heidelberg, Germany) generated isogenic BL lines. Cell lines for the screen were chosen with a focus on BL, other entities were introduced for comparison.

Cells were cultured in RPMI 1640 medium supplemented with 10–20% FBS, 1% Glutamine and 1% Penicillin/Streptomycin at 37 °C, at 5% CO_2_ with humidification. Cells with a passage number of 3–6 were used for drug screening. Cell lines were authenticated externally at Multiplexion GmbH (Immenstaad, Germany) by Single Nucleotide Polymorphism (SNP) profiling and by comparison of obtained signatures to reference signatures^58^.

### Drug library

For the single agent drug screen we built a library of 32 drugs, 10 concentrations each using threefold dilution to reach end concentrations from 30 µM to 1.5 nM. The range of concentrations allows for a wide range of viability effects, which is needed to generate IC50 and AUC. For the combination screen we built a library of 32 drugs, 10 concentrations each using threefold dilution to reach concentration ranges 30 µM–1.5 nM, 15 µM–0.76 nM or 10 µM–0.5 nM depending on drug potency. The choice of library drugs aimed at covering a wide range of key oncogenic pathways, especially those active in haematological malignancies. For easier translation of the results into the clinical setting only drugs that are currently in clinical use or in clinical trials were used.

We used a parallel approach probing three combination components: ibrutinib at 200 nM, idelalisib at 200 nM and OTX015 at 200 nM with the drug library. Single agent control was generated by combining drug library with solvent solution.

SD07 was provided by Prof. A. Mokhir, Erlangen, Germany^59^. The remaining drugs were obtained from Sigma-Aldrich (St. Louis, MI, USA), Enzo Life Sciences (Farmingdale, NY, USA) and Selleck Chemicals (Houston, TX, USA). Compounds were dissolved in DMSO at 0.1–50 mM and stored at −20 °C.

### CTG viability assay

Cell viability was determined using CellTiterGlo (CTG) ATP assay (Promega). Cells were seeded onto 384-well plates (Griner Bio One) at 1.5 × 10^3^–7 × 10^3^ cells per well depending on the growth rate and exposed to the drug library (10 µl) or the drug library combined with 200 nM ibrutinib, idelalisib or OTX015 (10 µl each) for 48 h; the final volume per well was 50 µl. We included DMSO and PBS controls to exclude DMSO toxicity effects. Cells were then incubated with 14 µl CTG reagent. Luminescence was measured after 20 minutes using a TECAN Microplate Reader (Tecan Group) with an integration time of 200 ms. Relative viability was determined by normalizing luminescence units for each well to DMSO control.

### Screen quality

Screen quality was assessed by an inter-experiment comparison of the data points common for both the single agent setup and the combination setup. The correlation coefficient (R^2^) between the datasets was 0.77 (Fig. S1). The intra-experiment reproducibility for the combination screen was 0.77 to 0.87 (correlation coefficient). The intra-experiment reproducibility for the combination screen was assessed based on two independent experiments repeated on 6 cell lines. Excellent correlation with correlation coefficients (R^2^) from 0.76 to 0.96 was shown (Fig. S2).

### 10 × 10 validation matrix

Individual drug combinations were validated in a 10 × 10 concentration block experiment on a 384-well format in duplicates. Cell lines were treated with a matrix of 10 × 10 concentrations (2-fold dilution) of two drugs; we determined optimal starting concentrations based on dose-response curves to those drugs: 1 µM for SNS-032, 10 nM for everolimus, 10 µM for MK-2206 and 10 µM for OTX015. Luminescence data from the CTG viability assay was normalized to DMSO controls and then bound between 0% and 100% by a second normalization.

### Cloning sgRNA constructs and generation of lentivirus particles

sgRNA was cloned into pLKO5.sgRNA.EFS.GFP plasmid vector (kindly provided by Benjamin Ebert, Addgene plasmid: #57822) followed by cloning protocol from elsewhere^60,61^. All sgRNA target sequences were extracted from Human Brunello CRISPR knockout pooled library (kindly provided by David Root and John Doench, Addgene #73178) except sgRNA target sequences for mCherry and Luciferase (see below). Lentivirus packaging protocol was adapted from elsewhere^61^. In brief, it was performed in HEK293T/17 cell line (kindly provided by Stefan Fröhling, DKFZ, Heidelberg, Germany) using 2^nd^

**Table:** 

ID	Forward	Reverse
Luciferase	CACCGGGTATAATACACCGCGCTAC	AAACGTAGCGCGGTGTATTATACCC
mCherry	CACCGCGCCCTCGATCTCGAACTCG	AAACCGAGTTCGAGATCGAGGGCGC

generation packaging plasmid, psPAX2 and envelop plasmid, pMD2.G (kindly provided by Didier Trono, Addgene plasmid: #12260, #12259, respectively).

### CRISPR/Cas9 GFP competition assay

Cells were infected with lentivirus particles in 6 well cell culture plate and were maintained at appropriate cell culture condition. Cells were analyzed using BD LSRII flow cytometry (BD bioscience, USA) to determine the percentage of GFP positive cells. Data obtained from FB LSRII was analyzed using FlowJo v.10 software (Tree Star, Ashland, OR, USA).

### Data evaluation and statistical analysis

Normalisation of the data and basic data analysis were performed with Microsoft Excel. Luminescence data was normalized to a mean of DMSO controls to obtain relative viability in per cent, which in turn was used for further analyses.

IC50 curves and bee swarm plot were generated using GraphPadPrism 5 software (Graphpad Software, Inc., La Jolla, CA, USA). For the IC50 curves a non-linear regression with viability data bound between 0 and 100% was used. This model:

1 $$y=\frac{100}{1+{10}^{x-{log}IC50}},$$

where x is a logarithm of dose concentration, assumes a standard slope factor of -1.0.

P values (two-tailed) were calculated using an unpaired parametric t-test.

Subsequent data analysis was performed in R statistical environment (version 3.1.3, https://www.r-project.org/).

IC50 values were recalculated using drm function from the package ‘drc’ with four-parameter log-logistic model:

2 $$y=c+\frac{d-c}{1+exp(b({log}(x)-\,{log}(IC50)))},$$

where y is the response, x is the concentration, c and d are the upper and the lower limits of response respectively and d is the slope around the point of inflection (which is the IC50 value). Area under the curve (AUC) values were calculated using auc function from the package ‘flux’ following the trapezoid integration rule.

Heatmaps of clustered analysis were generated using heatmap.2 function from the package ‘gplots’ with default parameters for rows and columns clustering (Euclidean distance measure, complete linkage hierarchical clustering). Correlation matrices were calculated using cor function and plotted using corrplot function (with complete linkage hierarchical clustering) from the ‘corrplot’ package.

Combination index (CI) was calculated using the formula:

3 $$CI=\frac{{C}_{A,50}}{I{C}_{50,A}}+\frac{{C}_{B,50}}{I{C}_{50,B}},$$

where IC50,A and IC50,B are IC50 values for individual library drugs; C A,50 and C B,50 are concentrations of drug A and B at which they were used in combination which lead to 50% viability^35^. IC50 values for this analysis stem from the drm calculation Combination indices for 10 × 10 matrices and isobologram analyses were performed using CompuSyn software (ComboSyn, Inc., Paramus, NJ, USA).

## Electronic supplementary material

Supplementary information

Dataset 1: Supplementary figures

Dataset 2: Supplementary tables

## Data Availability

All data generated or analysed during this study are included in this published article (and its Supplementary Information files).
