# Supplementary material for: Drug-based perturbation screen uncovers synergistic drug combinations in Burkitt lymphoma
Source: Sci Rep. 2018 Aug 13;8:12046. doi: 10.1038/s41598-018-30509-3 (PMC6089937; doi:10.1038/s41598-018-30509-3)
Supplement: Supplementary file 1 — Supplementary information [file 41598_2018_30509_MOESM1_ESM.pdf]

Supplementary information

Drug-based perturbation screen uncovers synergistic drug combinations in Burkitt lymphoma

Authors:

K. Tomska[1]*, R. Kurilov[2,3], KS. Lee[1], J. Hüllein[1], M. Lukas[1], L. Sellner[5], T. Walther[1], L. Wagner[1], M. Oleś[4], B. Brors[2], W. Huber[4], T. Zenz[1,6]

[1] Molecular Therapy in Haematology and Oncology & Department of Translational Oncology, NCT and DKFZ, Heidelberg, Germany
[2] Division of Applied Bioinformatics, DKFZ, Heidelberg, Germany
[3] Faculty of Biosciences, Heidelberg University, Germany
[4] Genome Biology Unit, EMBL, Heidelberg, Germany
[5] Department of Medicine V, University Hospital Heidelberg, Germany
[6] Dept. of Hematology, University Hospital and University of Zurich, Switzerland

Supplementary Figure 1. Evaluation of surrogate values for drug response: Comparison of IC50 and AUC. IC50 regression and AUC for compounds with variable viability effects in exemplary cell lines is shown. AUC provides a robust surrogate for nontoxic drugs or drugs with constant viability effect across the concentration range and was used for further analysis.

Supplementary Figure 2. Drug response based clustering of blood cancer cell lines. The analysis included all single agent data (n=42; grey indicates missing values). Adding additional cell lines to the analysis results in clustering similar to the one in Fig. 2, which supports the robustness of the identified clusters of response. In addition to drug response phenotypes shown in Fig. 2, a larger venetoclax sensitive subgroup (including Maver-1, Mino, HBL-2, Granta-519, DOGKIT, OCI-LY-1 and OCI-LY8) was identified. We show a BET inhibitor resistant line Awia, and as expected the CML cell line K562 is sensitive to dasatinib and saracatinib, targeting the BCR-ABL. Since drug libraries in both dataset did not overlap completely, viability data for missing values are marked grey.

Supplementary Figure 3. Impact of p53 on drug response.

A) Association of drug response and *TP53* mutation with the example of nutlin-3.
B) The comparison of isogenic cell lines with wild-type TP53 and knock-out of TP53 showed variable response to doxorubicin and nutlin-3. Relative viability of TP53 wild-type and TP53-deficient line Séraphine is shown in the bi plot. The outliers correspond to active concentrations of doxorubicin and nutlin-3 specific to wild-type p53 (marked blue and orange respectively).
C) Dose-response curves of TP53 wild-type and TP53-deficient Séraphine lines to different drugs. There were no significant differences in drug response to other drugs included in the screen, i.e. Src-family inhibitor AZD-0530 (saracatinib), Chk1/2 inhibitor AZD7762 or PI3K inhibitor idelalisib. No difference in response to the chemotherapeutic fludarabine was observed, while doxorubicin and nutlin-3 showed differential activity.

Supplementary Figure 4. Effects of ibrutinib and idelalisib combinations in BL cell lines. Scatter plots of viability data for combinations of idelalisib (x-axis) vs. ibrutinib (y-axis) with each library drug. Ibrutinib and idelalisib have very similar response patterns, as the data aligns along the diagonal. A shift from the diagonal indicates preferential combination effects.

Supplementary Figure 5. Cluster analysis of response to library drugs (combination drug screen) and their combination with ibrutinib, idelalisib and OTX015 in 18 BL lines. Unsupervised clustered analysis conducted on four parallel arms of the combination screen (DMSO, ibrutinib, idelalisib and OTX015) separately. The clustering of cell lines after the addition of ibrutinib (B) does not change compared to the single agent control (A), which again indicates low potency as a combination partner. The pattern of response to the combination with idelalisib (C) is very analogous to the one with ibrutinib, showing the similarity of those drugs. The fourth arm of the combinatorial setup results in a completely different pattern of response determined by the sensitivity to BETi by OTX015 (D).

Supplementary Figure 6. Assessing drug interaction of PI3K pathway inhibitor and BETi based on dose-response curves. Dose-response curves of idelalisib, MK-2206 and everolimus and their combinations with OTX015 in cell lines, where CI could not be determined (Gumbus, Salina, BL-41) show synergy,

as the response curve for the combination (green) lies below the single agent curves (red and blue) pointing towards stronger viability effect than expected.

Supplementary Figure 7. Assessing drug interaction based on dose-response curves. Exemplary assessment of drug interaction between Survivin inhibitor YM155 and BET inhibitor OTX015 if the combination curve (green) shows stronger effect on viability than single drugs alone (blue and red), synergy is assumed, i.e. Namalwa, DG75, Gumbus. If the combination curve (green) shows similar effect on viability compared to single drugs alone (blue and red), the combination is additive, i.e. Séraphine, Ramos, BL-60, BL-2, and LY47. If the combination curve (green) shows weaker effect on viability than one of the single drugs alone (blue and red), the combination is antagonistic, i.e. Raji, Salina, Yakobo, Awia, BL-41.

Supplementary Figure 8. Quality control of the drug response profiling platform.

A) Good reproducibility of two datasets presented, single agent drug screen (CLS) and combination drug screen (CCLS). The x-axis represents relative viability data points from the single agent drug screen (n=3740) and the y-axis shows the corresponding relative viability from the combination screen.
B) Good reproducibility within the single agent platform assessed by high correlation between two independent experiments on BL-41, SU-DHL-5 and Séraphine with R^2 values of 0.77, 0.85 and 0.87 respectively.
C) Good reproducibility within the combination drug screen was shown by high correlation between two independent experiments on BJAB, BL-7, BL-60, DOGKIT, Gumbus and Séraphine. BL-7 was the least robust cell line with a correlation coefficient R^2=0.76, while reproducibility in DOGKIT was excellent with a correlation coefficient R^2=0.96.

Supplementary Table S1. Single agent screen viability data.

Supplementary Table S2. Combination screen viability data.

Supplementary Table S3. Viability data from both data sets for all included cell lines and drugs.

Supplementary Table S4. List of cell lines.
