# Supplementary material for: Drug-based perturbation screen uncovers synergistic drug combinations in Burkitt lymphoma
Source: Sci Rep. 2018 Aug 13;8:12046. doi: 10.1038/s41598-018-30509-3 (PMC6089937; doi:10.1038/s41598-018-30509-3)
Supplement: Supplementary file 2 — Dataset 1: Supplementary figures [file 41598_2018_30509_MOESM2_ESM.pdf]

S1

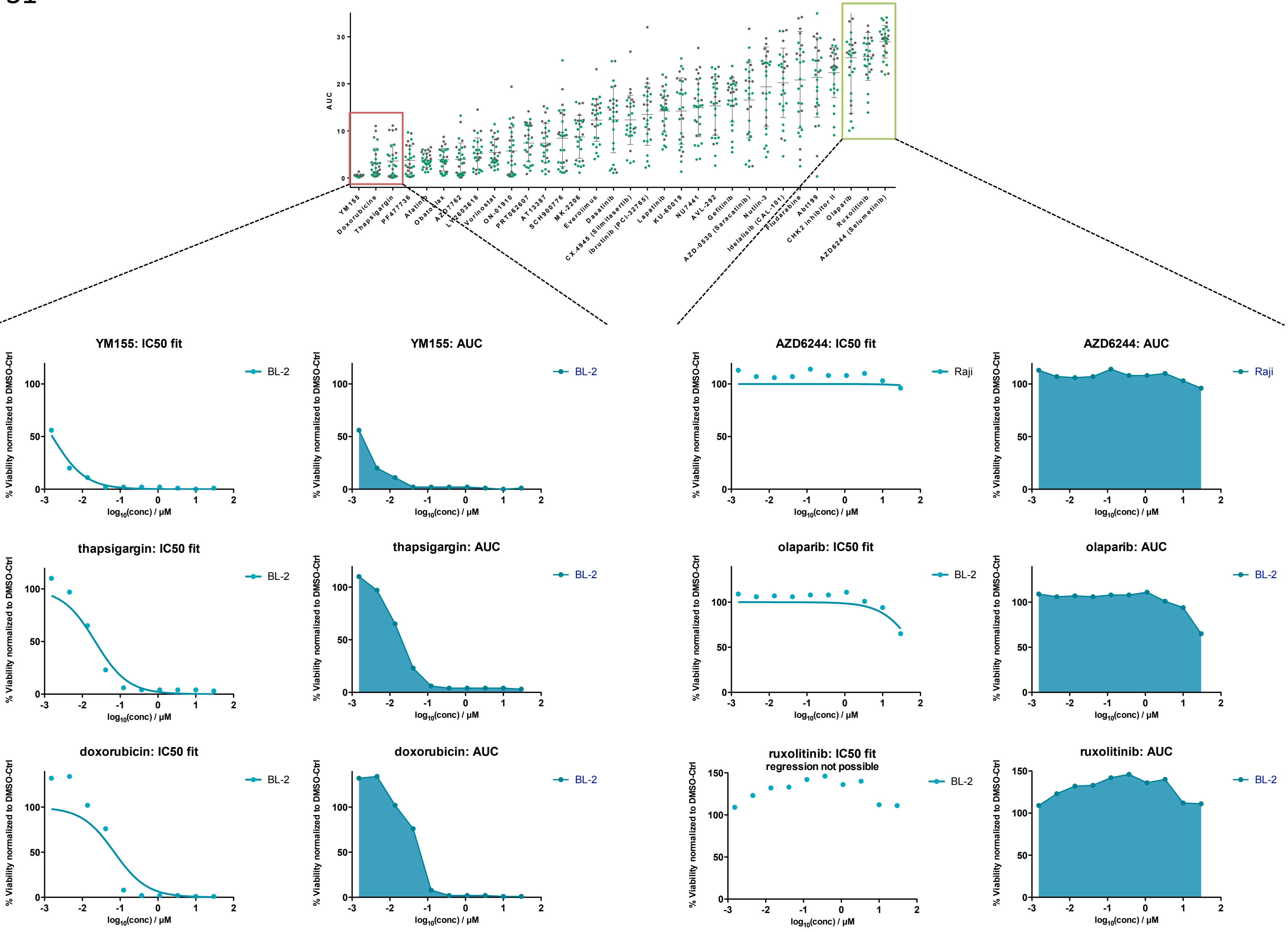
AUC
YM155
Doxorubicine
Thapsigargin
PF477736
Afatinib
Obatoclax
AZD7762
LY2603618
Vorinostat
ON-01910
PRT062607
AT13387
SCH900776
MK-2206
Everolimus
Dasatinib
CX-4945 (Silmitasertib)
Ibrutinib (PCI-32765)
Lapatinib
KU-60019
NU7441
AVL-292
Gefitinib
AZD-0530 (Saracatinib)
Idelalisib (CAL-101)
Nutlin-3
Fludarabine
Abt199
CHK2 inhibitor II
Olaparib
Ruxolitinib
AZD6244 (Selumetinib)
YM155: IC50 fit
YM155: AUC
AZD6244: IC50 fit
AZD6244: AUC
thapsigargin: IC50 fit
thapsigargin: AUC
olaparib: IC50 fit
olaparib: AUC
doxorubicin: IC50 fit
doxorubicin: AUC
ruxolitinib: IC50 fit
regression not possible
ruxolitinib: AUC
BL-2
Raji
% Viability normalized to DMSO-Ctrl
log10(conc) / µM

S2

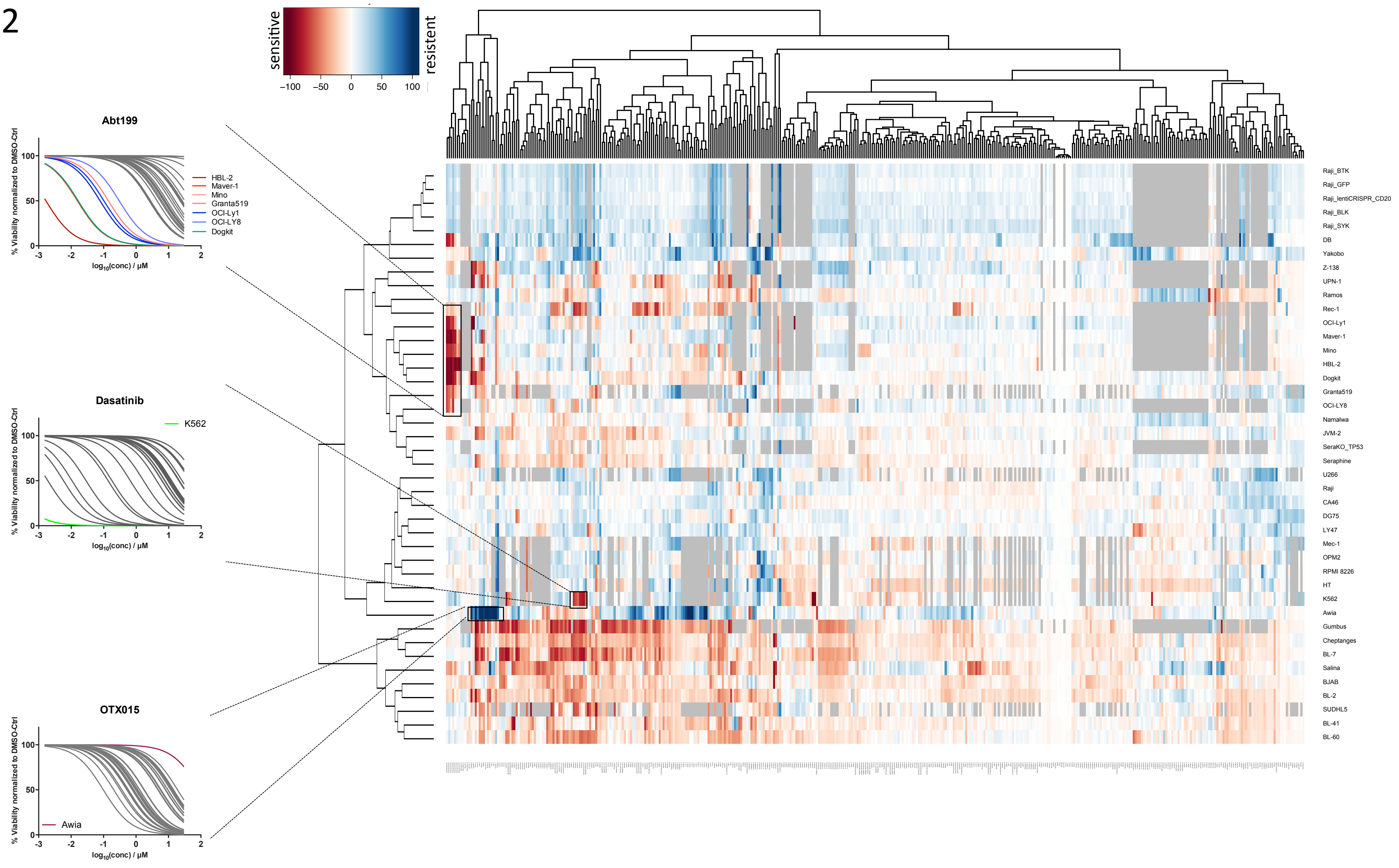
sensitive
resistent
−100
−50
0
50
100
Abt199
% Viability normalized to DMSO-Ctrl
log10(conc) / µM
HBL-2
Maver-1
Mino
Granta519
OCI-Ly1
OCI-LY8
Dogkit
Dasatinib
K562
OTX015
Awia
Raji_BTK
Raji_GFP
Raji_lentiCRISPR_CD20
Raji_BLK
Raji_SYK
DB
Yakobo
Z-138
UPN-1
Ramos
Rec-1
OCI-Ly1
Maver-1
Mino
HBL-2
Dogkit
Granta519
OCI-LY8
Namalwa
JVM-2
SeraKO_TP53
Seraphine
U266
Raji
CA46
DG75
LY47
Mec-1
OPM2
RPMI 8226
HT
K562
Awia
Gumbus
Cheptanges
BL-7
Salina
BJAB
BL-2
SUDHL5
BL-41
BL-60

S3

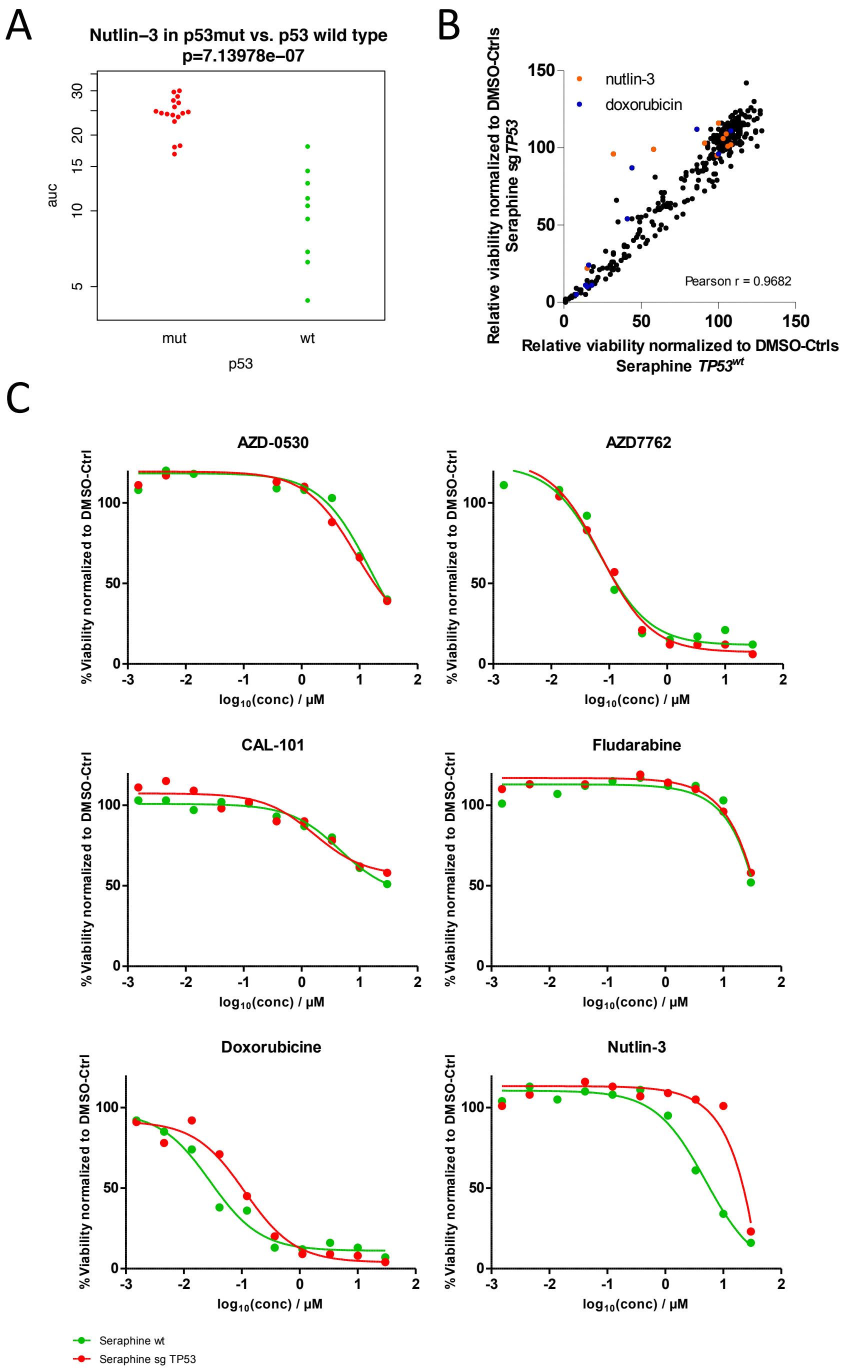
A
Nutlin–3 in p53mut vs. p53 wild type
p=7.13978e–07
auc
p53
mut
wt
B
nutlin-3
doxorubicin
Pearson r = 0.9682
Relative viability normalized to DMSO-Ctrls
Seraphine sgTP53
Relative viability normalized to DMSO-Ctrls
Seraphine TP53wt
C
AZD-0530
AZD7762
CAL-101
Fludarabine
Doxorubicine
Nutlin-3
% Viability normalized to DMSO-Ctrl
log10(conc) / µM
Seraphine wt
Seraphine sg TP53

S4

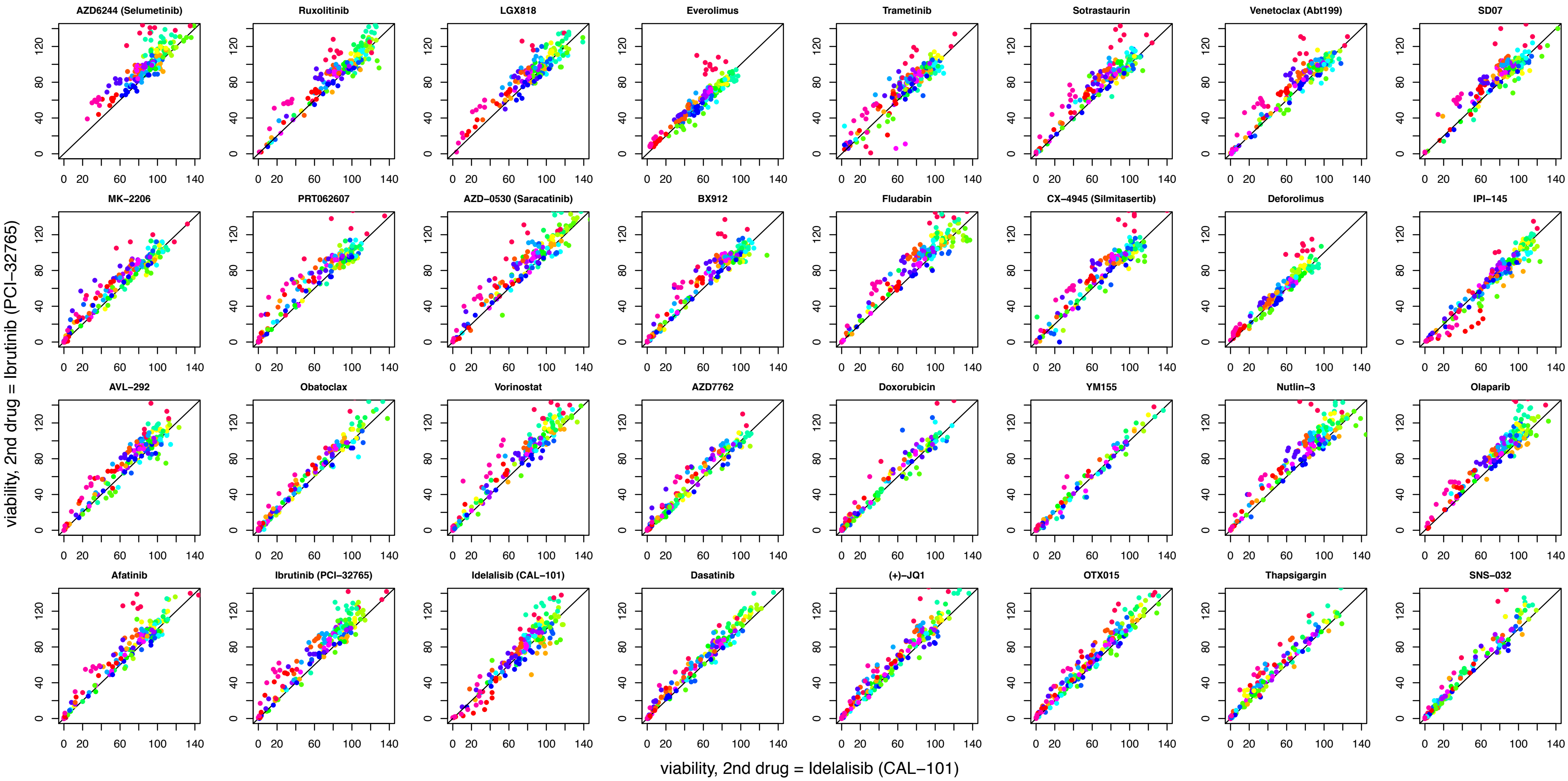
AZD6244 (Selumetinib)
Ruxolitinib
LGX818
Everolimus
Trametinib
Sotrastaurin
Venetoclax (Abt199)
SD07
MK−2206
PRT062607
AZD−0530 (Saracatinib)
BX912
Fludarabin
CX−4945 (Silmitasertib)
Deforolimus
IPI−145
AVL−292
Obatoclax
Vorinostat
AZD7762
Doxorubicin
YM155
Nutlin−3
Olaparib
Afatinib
Ibrutinib (PCI−32765)
Idelalisib (CAL−101)
Dasatinib
(+)−JQ1
OTX015
Thapsigargin
SNS−032
viability, 2nd drug = Ibrutinib (PCI−32765)
viability, 2nd drug = Idelalisib (CAL−101)

S5

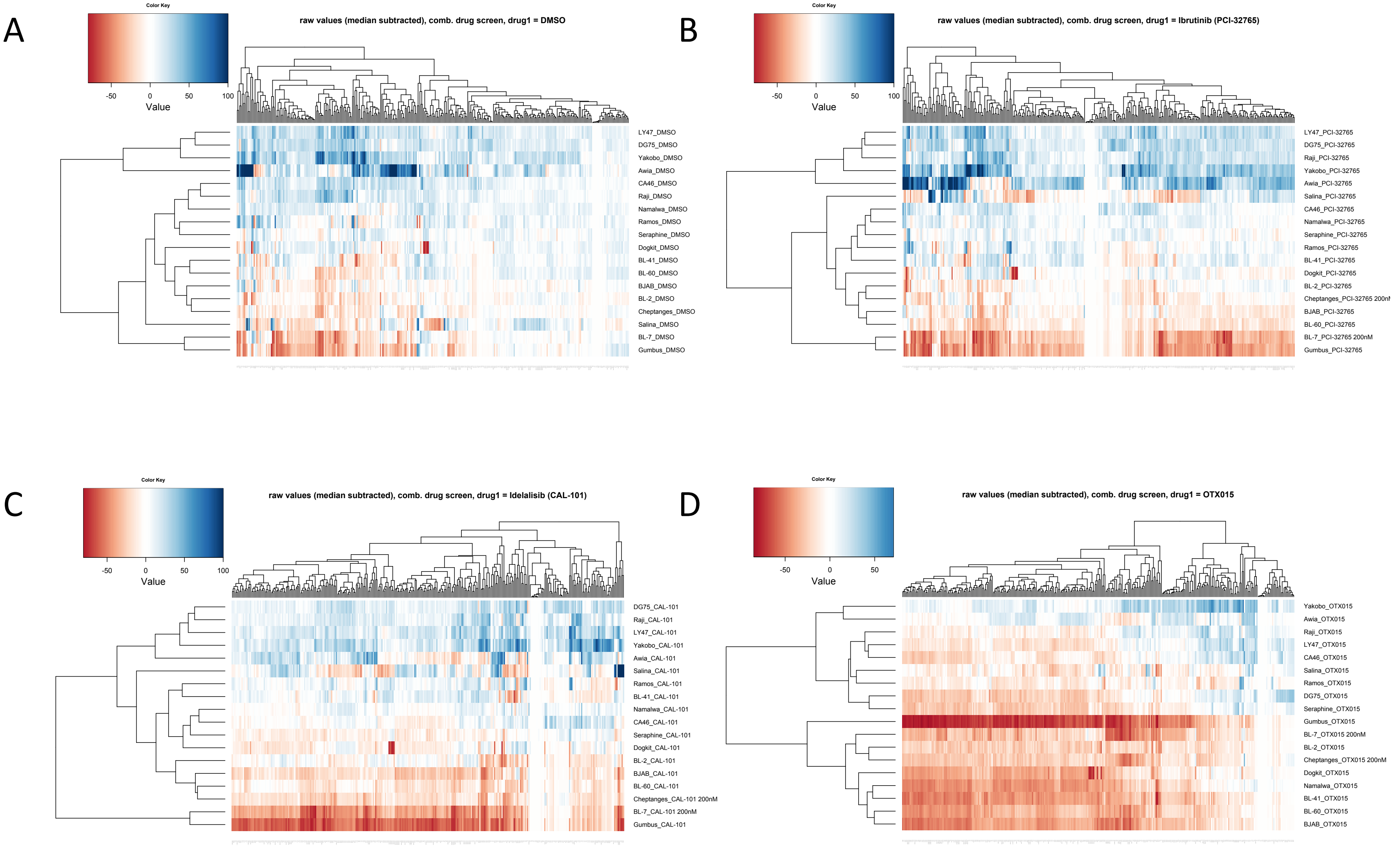
A
Color Key
-50
0
50
100
Value
raw values (median subtracted), comb. drug screen, drug1 = DMSO
LY47_DMSO
DG75_DMSO
Yakobo_DMSO
Awia_DMSO
CA46_DMSO
Raji_DMSO
Namalwa_DMSO
Ramos_DMSO
Seraphine_DMSO
Dogkit_DMSO
BL-41_DMSO
BL-60_DMSO
BJAB_DMSO
BL-2_DMSO
Cheptanges_DMSO
Salina_DMSO
BL-7_DMSO
Gumbus_DMSO
B
Color Key
-50
0
50
100
Value
raw values (median subtracted), comb. drug screen, drug1 = Ibrutinib (PCI-32765)
LY47_PCI-32765
DG75_PCI-32765
Raji_PCI-32765
Yakobo_PCI-32765
Awia_PCI-32765
Salina_PCI-32765
CA46_PCI-32765
Namalwa_PCI-32765
Seraphine_PCI-32765
Ramos_PCI-32765
BL-41_PCI-32765
Dogkit_PCI-32765
BL-2_PCI-32765
Cheptanges_PCI-32765 200nI
BJAB_PCI-32765
BL-60_PCI-32765
BL-7_PCI-32765 200nM
Gumbus_PCI-32765
C
Color Key
-50
0
50
100
Value
raw values (median subtracted), comb. drug screen, drug1 = Idelalisib (CAL-101)
DG75_CAL-101
Raji_CAL-101
LY47_CAL-101
Yakobo_CAL-101
Awia_CAL-101
Salina_CAL-101
Ramos_CAL-101
BL-41_CAL-101
Namalwa_CAL-101
CA46_CAL-101
Seraphine_CAL-101
Dogkit_CAL-101
BL-2_CAL-101
BJAB_CAL-101
BL-60_CAL-101
Cheptanges_CAL-101 200nM
BL-7_CAL-101 200nM
Gumbus_CAL-101
D
Color Key
-50
0
50
Value
raw values (median subtracted), comb. drug screen, drug1 = OTX015
Yakobo_OTX015
Awia_OTX015
Raji_OTX015
LY47_OTX015
CA46_OTX015
Salina_OTX015
Ramos_OTX015
DG75_OTX015
Seraphine_OTX015
Gumbus_OTX015
BL-7_OTX015 200nM
BL-2_OTX015
Cheptanges_OTX015 200nM
Dogkit_OTX015
Namalwa_OTX015
BL-41_OTX015
BL-60_OTX015
BJAB_OTX015

S6

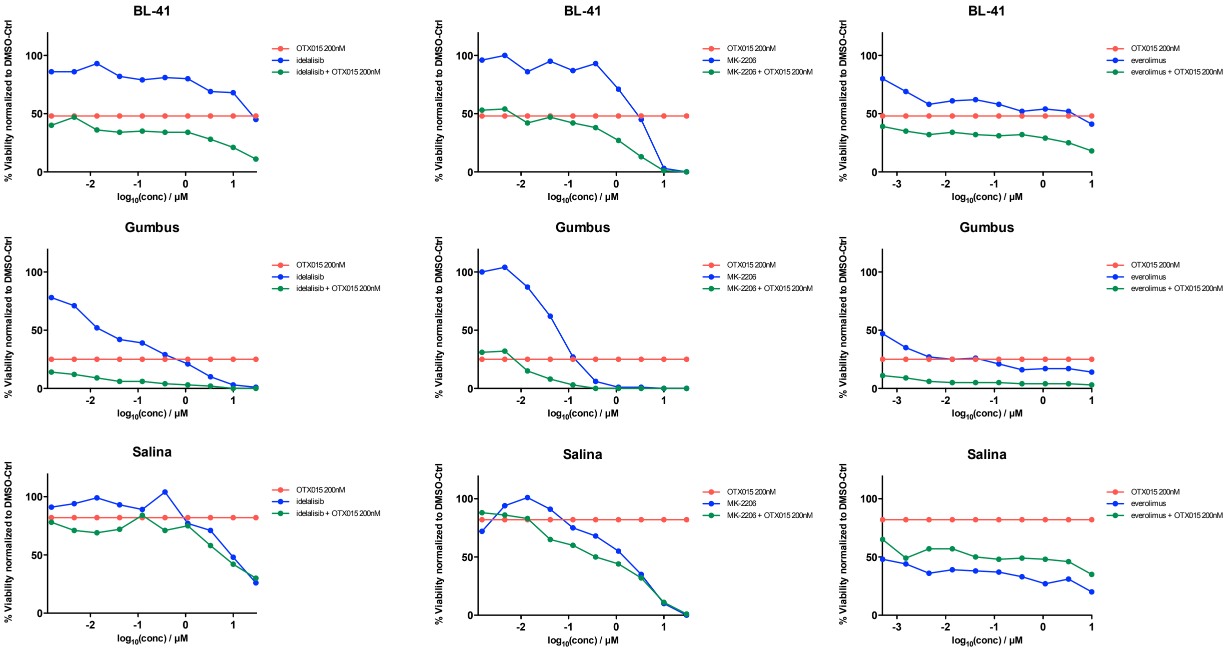
BL-41
Gumbus
Salina
% Viability normalized to DMSO-Ctrl
log10(conc) / µM
OTX015 200nM
idelalisib
idelalisib + OTX015 200nM
MK-2206
MK-2206 + OTX015 200nM
everolimus
everolimus + OTX015 200nM

S7

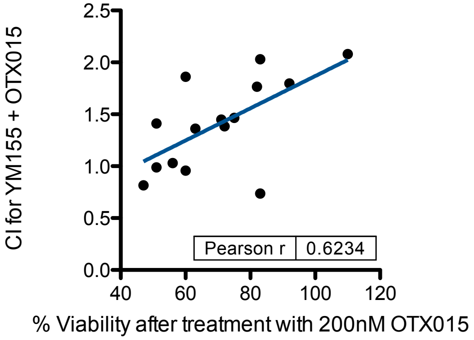

CI for YM155 + OTX015
2.5
2.0
1.5
1.0
0.5
0.0
40
60
80
100
120
Pearson r
0.6234
% Viability after treatment with 200nM OTX015

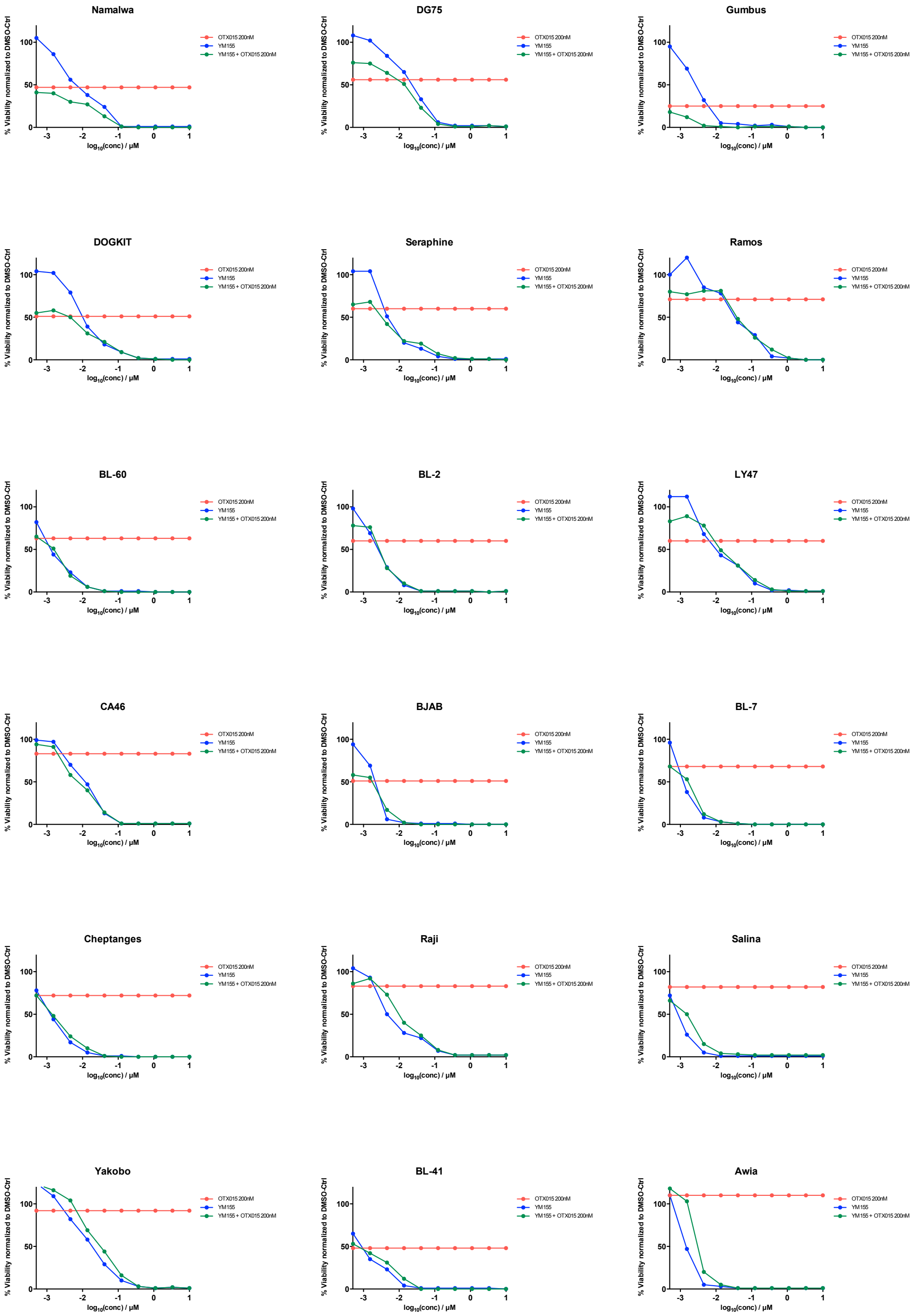

Namalwa
DG75
Gumbus
DOGKIT
Seraphine
Ramos
BL-60
BL-2
LY47
CA46
BJAB
BL-7
Cheptanges
Raji
Salina
Yakobo
BL-41
Awia
OTX015 200nM
YM155
YM155 + OTX015 200nM
% Viability normalized to DMSO-Ctrl
$log_{10}(conc) / \mu M$

S8

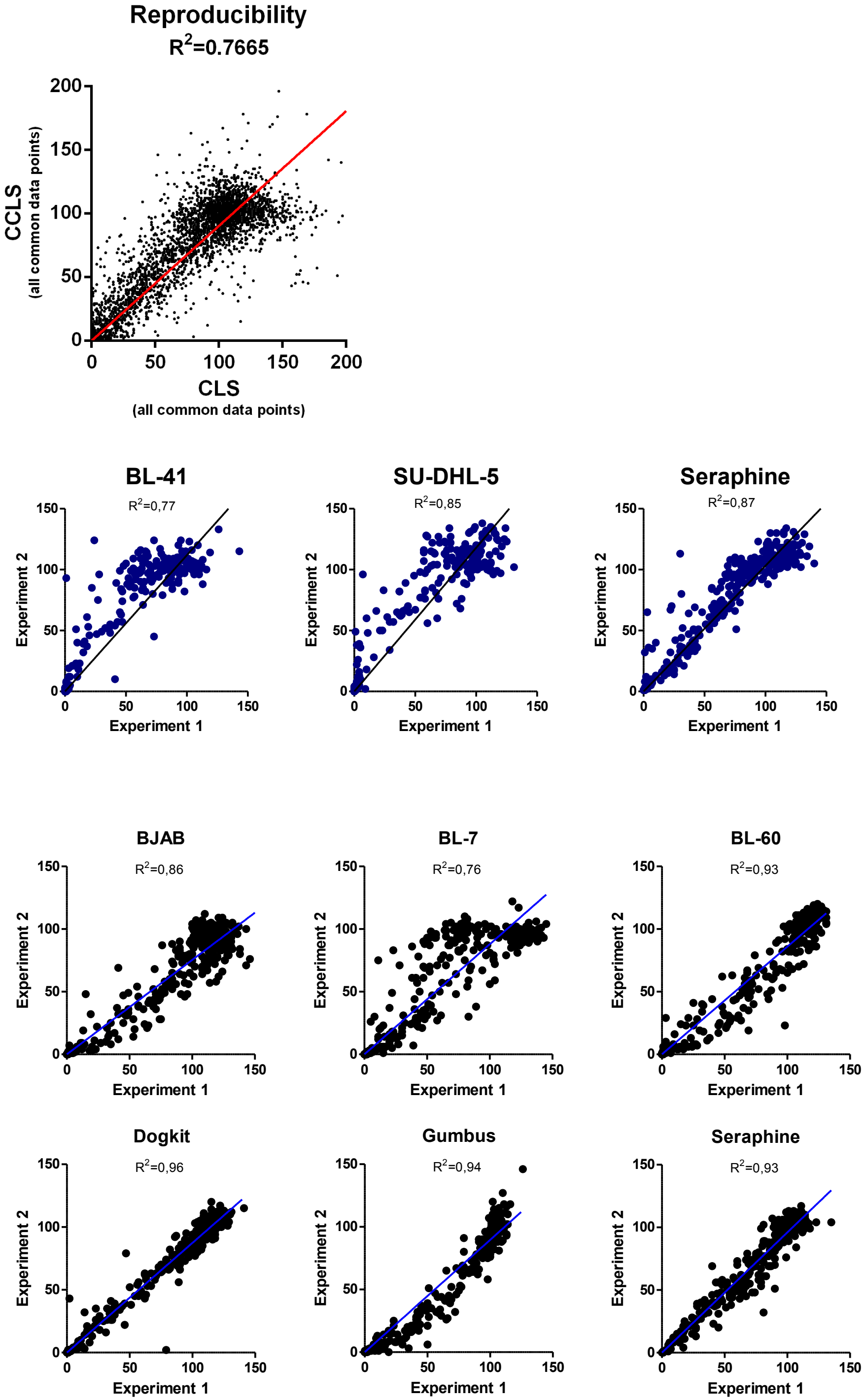
Reproducibility
R^2=0.7665
CCLS
(all common data points)
CLS
(all common data points)
0
50
100
150
200
BL-41
R^2=0,77
SU-DHL-5
R^2=0,85
Seraphine
R^2=0,87
BJAB
R^2=0,86
BL-7
R^2=0,76
BL-60
R^2=0,93
Dogkit
R^2=0,96
Gumbus
R^2=0,94
Seraphine
R^2=0,93
Experiment 1
Experiment 2
